# “My Autism is Linked with Everything”: at the Crossroads of Autism and Diabetes

**DOI:** 10.1007/s10803-023-06033-3

**Published:** 2023-07-22

**Authors:** Ritwika Vinayagam, Christopher Tanner, David Harley, Shamshad Karatela, Katie Brooker

**Affiliations:** 1 Queensland Centre for Intellectual and Developmental Disability (QCIDD), Mater Research Institute-UQ, The University of Queensland, Level 2, 39 Annerley Rd, South Brisbane, QLD 4101 Australia; 2 The Cooperative Research Centre for Living with Autism (Autism CRC), Level 3, Foxtail Building UQ Long Pocket Campus, 80 Meiers Rd, Indooroopilly, QLD 4068 Australia; 3 UQ Centre for Clinical Research, Royal Brisbane & Women’s Hospital Campus, Building 71/918, Herston, QLD 4029 Australia; 4 School of Pharmacy, The University of Queensland, Level 4/20 Cornwall St, Woolloongabba, QLD 4102 Australia; 5 Australian Institute of Tropical Health and Medicine (AITHM), James Cook University, Building 48 1 James Cook Drive, Douglas, QLD 4811 Australia

**Keywords:** Autism, Diabetes, Self-Management, Chronic Disease, Autistic Burnout

## Abstract

Autistic adults experience stark health disparities and difficulties accessing health care. Their realities of managing complex health conditions are unknown. Our research explored the experience of Autistic adults self-managing diabetes. Interviews with Autistic adults with diabetes and their support people were thematically analysed to identify three key themes. The Autistic experience influenced diabetes self-management, including autism-unique challenges and strengths. Participants prioritised avoiding Autistic burnout over diabetes self-management; mitigating the psychosocial pressures of neurotypical systems took precedence. Health professionals often separated autism and diabetes subsequently overlooking key factors impacting diabetes self-management. To better meet the needs of Autistic adults, diabetes care and health management more broadly should be considered within the context of autism, including supports for self-management during Autistic burnout.

Autism is a lifelong condition associated with social communication and social interaction differences, and restricted or repetitive behaviours and interests (American Psychiatric Association, 2013). Emerging evidence suggests diabetes is more prevalent in Autistic adults^1^ than in the general population (Chen, et al., 2016; Shedlock, et al., 2016). Current diabetes and autism research has focused on prevalence (Chen, et al., 2016; Kohane, et al., 2012; Shedlock, et al., 2016), neglecting the lived experience. Given the increased risk of mortality from chronic conditions experienced by Autistic adults (Hirvikoski, et al., 2016; Hwang, et al., 2019), understanding how Autistic experiences potentially influence diabetes and other chronic health conditions is crucial.

The primary approach to diabetes is self-management (Shrivastava, et al., 2013). This positions individuals as active participants and experts in their diabetes management. Diabetes self-management is focused on maintaining blood glucose levels within a target range. Diabetes self-management tasks differ between type 1, type 2, and gestational diabetes as well as between individuals but can include: checking blood glucose levels multiple times a day, taking medication (e.g., insulin injections or using an insulin pump, oral medications), calculating insulin dosages, counting carbohydrates, and considering the impact of food and exercise on blood glucose levels. Additional considerations are needed around sick days, pregnancy, and health screening. People living with diabetes, like everyone, should maintain a healthy diet and regular physical activity (Shrivastava, et al., 2013). Diabetes requires a high level of engagement with the health system; people living with diabetes require regular health checks with multiple health professionals, filling prescriptions, and buying diabetes supplies (e.g., testing strips for glucose monitor, needles) (Harris, 2000). In Australia, subsidies exist to ensure people with diabetes have afforded access to diabetes supplies and medications however out of pocket expenses can still be prohibitive. Optimal diabetes self-management is important to reduce the risk of diabetes complications, including diabetic kidney disease, diabetic retinopathy and amputations (Stolar, 2010).

Implementing diabetes self-management tasks into practice is complex. The practicalities of self-managing diabetes require significant time, planning and forethought, and financial considerations which contribute towards an additional cognitive and mental load (Alexandre, et al., 2021; Stuckey & Peyrot, 2020). Additionally, individuals report feeling burdened by their fear of diabetes related complications, societal stereotypes of diabetes and the feeling of otherness living with diabetes causes (Sibounheuang, et al., 2020). Poor attitudes towards diabetes by health professionals as well as communication between individuals and health professionals further impacts diabetes self-management, as health advice often fails to account for the complex nuances of day-to-day lived experience (Nam, et al., 2011). Collectively, this contributes to reduced ability to engage in diabetes self-management (Stuckey & Peyrot, 2020).

There is no research on Autistic adults’ experiences of self-managing diabetes, as such, an autistic co-author of this paper has included a reflection in the hope of illustrating their personal experience of living with autism and having a chronic disease (Fig. 1). As there is no academic literature to draw on, it is useful to consider more broadly the unique challenges and strengths which impact self-management experiences. Preference for routine, sensory sensitivities, masking, and Autistic burnout present unique aspects of the Autistic experience which may influence diabetes self-management. The preference for routine may assist individuals in managing medications (Gardner, 2015). Conversely, other Autistic influences such as masking, where Autistic people hide aspects of themselves or their behaviours to conform to social norms, may lead health professionals to overlook the impacts of autism-specific characteristics on diabetes self-management (e.g., sensory needs around food and diet). Sensory sensitivities around using diabetes related technology, which often involves wearing adhesive products and filaments under the skin for extended periods, may be challenging.

**Fig. 1:**
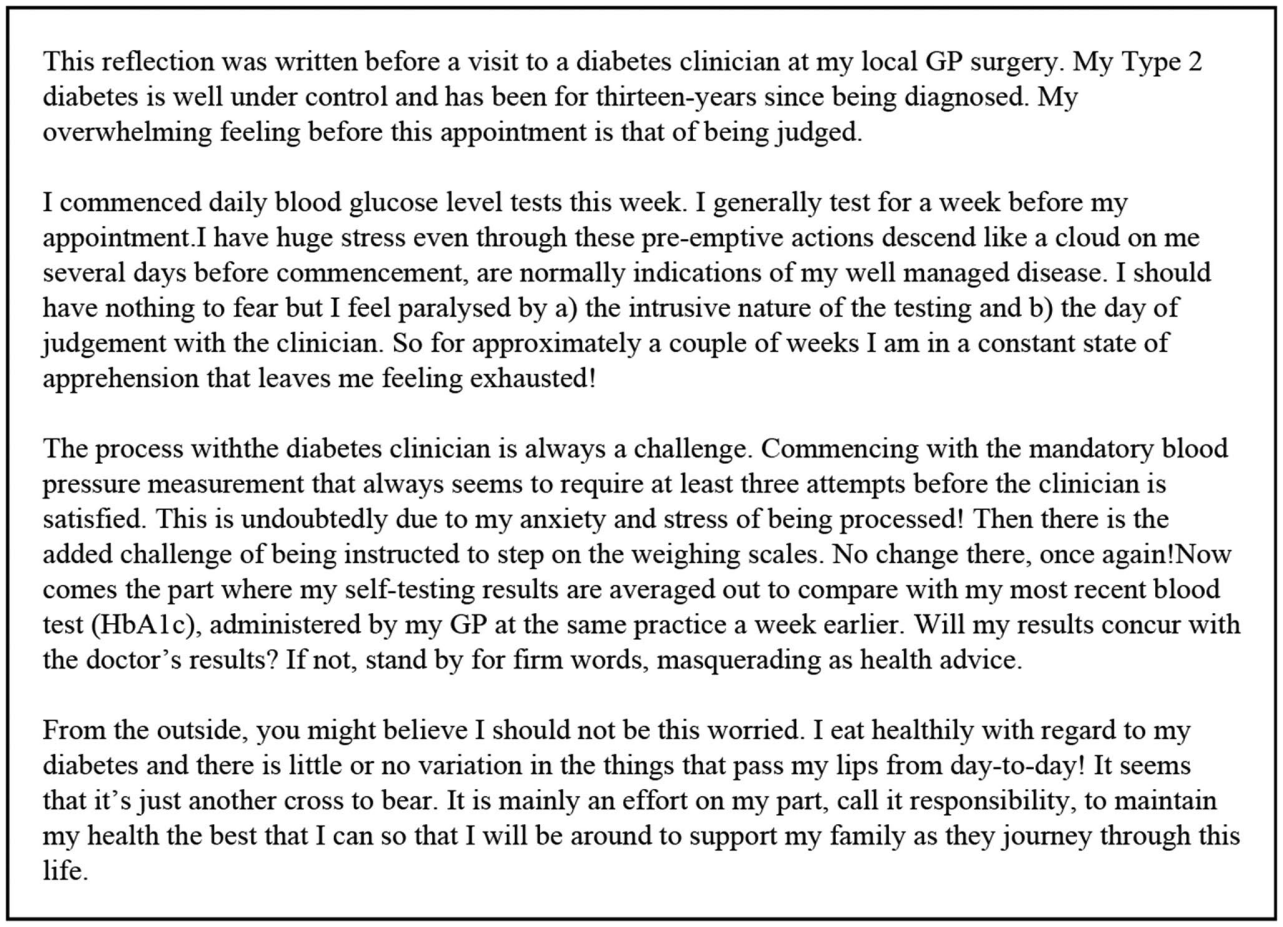
An Autistic Author’s reflections on the Autistic experience of Self-managing Diabetes

Autistic burnout results “from chronic life stress and a mismatch of expectations and abilities without adequate supports” and is pervasive (Raymaker, et al., 2020, p. 133). Research into Autistic burnout remains extremely limited despite being well-known within Autistic communities. Autistic burnout is caused by a range of factors, most prominently the challenges associated with living in systems and environments insensitive to Autistic needs (Higgins, et al., 2021; Raymaker, et al., 2020). Emerging research details its influences on psychosocial aspects, including chronic exhaustion, loss of functional skills, and social withdrawal (Higgins, et al., 2021; Raymaker, et al., 2020). Given the reduced ability to engage in self-care during Autistic burnout, it is possible that its associated influences may negatively impact diabetes self-management (Higgins, et al., 2021; Raymaker, et al., 2020).

Accessible health care and health services are important aspects of diabetes self-management. Autistic adults experience numerous barriers to accessing healthcare. Health professionals lack autism knowledge which impacts the quality and appropriateness of health care Autistic adults receive (Bradshaw, et al., 2019; Mason, et al., 2019). Further, Autistic people have sensory needs which are often overlooked in healthcare environments commonly characterised by bright lighting, strong smells, and unpredictable loud noises all contribute towards sensory overload (Bradshaw, et al., 2019; Mason, et al., 2019). Differences in communication between healthcare providers and Autistic people, for example healthcare professional’s use of open-ended questions, may also impact the quality of healthcare due (Bradshaw, et al., 2019; Mason, et al., 2019). As research in the general population indicates, patient-to-provider relationships form a vital aspect of good diabetes outcomes (Nam, et al., 2011). It is therefore crucial to understand the interaction of these factors on how, and whether individuals access the multi-disciplinary healthcare required for good diabetes self-management.

Given the lack of understanding of the Autistic experience in the context of diabetes self-management and health more broadly, we address the question, ‘What is the lived experience of Autistic adults when self-managing diabetes?’. Through exploring this question, we aimed to:

- Develop an understanding of the barriers to diabetes self-management experienced by Autistic adults.
- Identify how health professionals can better support Autistic adults in self-managing diabetes.

## Methods

A qualitative, phenomenological perspective guided our exploration of the lived experiences of Autistic people living with diabetes. A qualitative approach was best suited to this research question as it is a novel area of research (Kleiman, 2004). We worked with an Autistic researcher (CT) to ensure our methods were sensitive to the needs of Autistic adults and to develop our research direction and data analysis. We used in-depth interviews to explore lived experience; the mostly unguided nature of interviews enabled us to encourage participants to reflect on their realities managing and living with diabetes by positioning them as the experts (Dicicco-Bloom & Crabtree, 2006). The flexibility conferred by interviews allowed exploration of avenues and insights offered by participants, not possible with a quantitative approach.

### Methodology

Phenomenological research emphasises the commonalities across individual experiences and understanding the contexts and intricacies underlying the lived experiences (Kleiman, 2004). This provided the flexibility to explore the heterogeneous experiences of autism (Geschwind, 2009) and consequently diabetes self-management. Transcendental phenomenology, a descriptive approach that places participant experiences and the meanings participants attach to them at the forefront (Davidsen, 2013), informed our data collection and analysis. As non-Autistic researchers, we believed the research objectivity underpinning transcendental phenomenology was imperative to accurately representing each Autistic individual’s experiences. Moreover, as non-Autistic researchers without lived experience of diabetes, we were not positioned to undertake an interpretative analysis approach given the considerable potential for misinterpretation.

### Participatory Approach

We aimed to establish a research advisory group to provide input into all stages of the research, however, this was impacted by the COVID-19 pandemic. We worked closely with one Autistic researcher (CT) who informed the development of the research direction, methods, and interpretation of results. We aimed to foster a collaborative approach to ensure our research was relevant and valid to Autistic individuals living with diabetes (Cargo & Mercer, 2008). Given that the Autistic community has voiced concerns regarding the disconnect in priorities between researchers and Autistic people (Pellicano, et al., 2014), it was essential that the Autistic researcher provided input across as much of the research process as possible. A participatory approach enabled us to capture subtleties that were not apparent to us as non-Autistic researchers without lived experience of autism and diabetes (Cargo & Mercer, 2008).

### Ethics Approval

Researchers obtained ethical and governance clearance through the Mater Human Research Ethics Committee (approval #59,383) and the University of Queensland Human Research Ethics Committee (approval #2,020,000,461). All participants provided informed consent.

### Recruitment

Convenience sampling was used to recruit participants by advertising the study on the social media platforms and newsletters of disability and autism organisations throughout Australia. Individuals were eligible for inclusion if they self-reported diagnoses of autism and type 1 diabetes or type 2 diabetes (hereafter referred to as participants), or self-reported they were a support person of an Autistic adult living with diabetes (hereafter referred to as support person), were over 18 years, lived in Australia and were able to provide informed consent. No exclusions were made based on intellectual disability.

Participants were screened for inclusion upon establishing contact with the research team. Authors emailed eligible individuals a participant information and consent form in Easy English or plain English, as appropriate to their communication preferences and needs. 10 individuals contacted researchers regarding participation, eight were involved in the final study.

### Data Collection

Data were collected from July to October 2020. Participants completed a short online survey through Qualtrics (Qualtrics, 2020) to collect participants’ demographics, communication preferences, and diabetes diagnosis. Researchers used the information from surveys to tailor the interview style and format to participants’ preferences, including any accommodations required to facilitate a positive interview experience (e.g., sending the interview guide before the meeting, asking clear and non-abstract questions).

The first author interviewed participants through video-calls (*n* = 6) and emails (*n =* 2) following a semi-structured interview guide. Interviews lasted from 56 to 114 minutes. Face-to-face interviews were not possible as data collection occurred during the COVID-19 pandemic. The interview guide, developed in collaboration between first, second and last authors, included questions on diabetes self-management, including day-to-day self-management activities (e.g., medication, diet, and physical activity), accessing healthcare for diabetes, and mental health. The interview guide was reviewed following each interview, and additional questions and prompts were added to promote reflexivity and ensure the interview questions reflected new insights gained post-interviews (Hsiung, 2008). Interviews were audio-recorded with participants’ consent, and the interviewer took brief handwritten notes during interviews. Video interviews lasted one to two hours and were transcribed by the first author. All interview and survey data were de-identified and labelled using unique participant codes. Data were stored on password-protected drives.

The first author received training in interviewing Autistic individuals from the research team. Strategies were used to ensure participant comfort during interviews, including adapting the delivery of the interview to participants’ preferences by offering video, telephone, email, or instant-chat interviews, and asking participants about their communication preferences (e.g. open-ended or specific questions and making adjustments to the interview style based on these preferences), and building rapport with participants before initiating interviews (Harrington, et al., 2014).

### Data Analysis

Transcripts and interview notes were analysed using thematic analysis to identify patterns in the lived experience of Autistic adults self-managing diabetes (Braun & Clarke, 2006). In line with our transcendental phenomenological approach, thematic analysis was approached on a mostly semantic level to sustain the focus on providing rich descriptions of participants’ experiences of living with and self-managing diabetes, and to maintain researchers’ objectivity (Boyatzis, 1998; Davidsen, 2013). A mix of inductive and deductive approaches were used. NVivo12 was used for data analysis (QSR International Pty Ltd, 2020).

The method used by Braun and Clarke (2006) informed the thematic analysis. The first author immersed themselves in the data by listening to audio-recordings of interviews while transcribing, and reading interview transcripts multiple times (Braun & Clarke, 2006). The first author systematically coded each interview. Uncertainties regarding the first author’s understanding of participants’ experiences were resolved through discussions with the last author (Mays & Pope, 2000). Upon coding all interviews, the first author reviewed the codes to identify similarities between codes (Braun & Clarke, 2006). Codes sharing patterns or meanings were collated into broader groups (undefined themes) and smaller groups within these themes (sub-themes). Both the first and last author reviewed the preliminary themes to identify if themes were sufficiently supported by data, to determine if themes were better classified as sub-themes, and if themes should be separated or merged (Braun & Clarke, 2006). The coded extracts were then reviewed under each theme to identify if the extracts from the interview were coherent with the theme (Braun & Clarke, 2006). Following this, interviews were reread to ascertain whether the synthesised themes were relevant within the context of *all* interviews and participant experiences, and to identify codes missed during previous coding (Braun & Clarke, 2006). The Autistic researcher provided input and insight on whether the non-Autistic researchers’ interpretation and coding of the data were consistent with Autistic experience. Co-jointly, the Autistic and non-Autistic researchers finalised the themes.

### Rigour

We promoted validity and rigour by using measures to minimise researcher biases and risks of mis-interpreting data (Mays & Pope, 2000). Team members separately coded data, with coded transcripts continuously shared throughout the analysis to resolve discrepancies and enrich interpretation. Member checking, by reviewing a selection of codes and discussing results with the Autistic researcher, was used to improve data validity and ensure the interpretations of non-Autistic researchers were reflective of the Autistic experience. (Mays & Pope, 2000). The first author engaged in regular peer-debriefings and maintained a reflexive research diary during data collection and analysis (Noble & Smith, 2015). These processes minimised the influences of any pre-conceptions and biases by raising awareness of and discussion around thought processes (Mays & Pope, 2000; Noble & Smith, 2015).

## Results

We interviewed seven Autistic people living with diabetes and one support person, see Table 1. Most participants had type 2 diabetes and had received an autism diagnosis in adulthood. None of the participants had an intellectual disability. The support person was also Autistic.

Each participant’s experience of autism and diabetes self-management was different from the others. Diet and physical activity were not at the forefront of their diabetes self-management, with participants mostly interested in speaking about healthcare access and facets of their life related to autism rather than diabetes.

**Table 1:** Participant Demographics

	Participants^*^ (*n* = 7)	Support People* (*n* = 1)
Gender (Female)	4 (57.1)	1 (100)
Age in years	44.6 (21–70)	35
Age in years at Autism Diagnosis	41.1 (17–66)	34
Years Since Autism Diagnosis	3.4 (0–8)	1
**Type of Diabetes**		N/A
**Type 1**	3 (42.9)	
**Type 2**	4 (57.1)	
**Years Living with Diabetes**	12.1 (4–25)	N/A
Ethnicity		
Caucasian	6 (85.7)	1 (100)
Asian	1 (14.3)	
Highest Level of Education		
High School	5 (71.4)	
TAFE	1 (14.3)	1 (100)
University	1 (14.3)	

^*^N (%) for categorical variables, mean (range) for continuous variables

### Autism has Distinct Influences on Diabetes self-management

Participants brought invaluable personal knowledge of autism in a neurotypical world to their diabetes self-management. They described the influences of autism on their day-to-day self-management of diabetes.

Executive functioning caused some participants to experience difficulties in remembering, planning, and completing diabetes self-management tasks such as taking medications, organising and attending diabetes appointments, and eating healthy diets (with its associated challenges of meal preparation):

*“Sometimes I wonder if I took my insulin and in my head I can recall standing in a certain place and injecting myself but I can’t be sure which day I’m remembering...” (female, type 1, mid-40s).*

Some participants who had challenges with executive functioning drew on the support of others to assist with the organisation of medical appointments:

*“[My wife] helped me get organised, she helped me get appointments, made me stick to my appointments, with my diabetes appointments. Always made sure that I had enough insulin.” (male, type 1, mid-30s).*

The interconnectedness between executive functioning, sensory overload and self-management was a “huge light bulb moment” during the reflective processes of the interview for a participant who was struggling with testing blood glucose levels and remembering to take insulin:

*“My autism is linked with everything and I should be thinking about it more often. It’s much more helpful knowing it and then I can be more aware of it when I’m [looking after my diabetes]...” (female, type 1, early-20s).*

Other aspects of the Autistic experience, including sensory sensitivities and preference for routine, created difficulties in adapting to diabetes self-management tasks. Participants, without health professional’s support, implemented strategies to address their challenges:

*“I would eat the same thing every day if I could, and the foods I like are not considered healthy. I hate the texture of cooked vegetables, so I must make soups to make it palatable.” (female, type 2, mid-40s).*

However, for one participant, their preference for “uniformity and compliance” (male, type 2, early 70s) drove them to attend diabetes appointments despite facing barriers such as unaccommodating sensory environments within healthcare settings.

Participants described regularly masking around their health professionals. Participants felt uncomfortable raising the impacts of autism on diabetes self-management with their health professional. In these instances, they masked by accepting advice that was unsuitable to their needs, and by “giving up” (male, type 2, mid-40s) on receiving diabetes care. They also withheld information on the influences of autism on functioning, diabetes self-management, and processing diabetes advice.

*“That’s why I don’t have the best management now because I’m not very upfront about my autism. I don’t feel comfortable talking about it with [health professionals] since they never mention my autism whenever my endocrinologist says anything I just go with the flow.” (female, type 1, early-20s)*

Participants who received an autism diagnosis after their diabetes diagnosis, found it strengthened diabetes self-management by validating and explaining the challenges to self-management they experienced. For one participant, their autism diagnosis provided them with the confidence to stop masking around health professionals and communicate their learning preferences. This produced changes in their understanding and implementation of complex diabetes advice such as changes to their insulin doses.

*“I do ask more questions [now], so if I feel like they’re going too fast…I’ll tell them to slow down, and just ask open-ended questions just so I can get a fuller, a better and fuller understanding of what they’re trying to tell me with my diabetes.” (male, type 1, mid-30s)*

### Prospect of Autistic Burnout Means Diabetes is a Lower Priority

Autism emerged as a higher priority than diabetes. All participants described experiences of Autistic burnout, which was often caused by unsupportive environments:

*“…the world makes Autistic people sick in essence because we’re expected to be someone who we’re not.” (female, type 2, late-40s).*

Participants were cognisant of avoiding Autistic burnout as it impacted all facets of their life, including their ability to self-manage their diabetes:

*“Being on the Autistic spectrum, there’s a lot more grievous things for me to think about, than there is diabetes, to be honest.” (male, type 2, early-70s).*

Participants’ and health professionals’ expectations of diabetes self-management contributed to Autistic burnout:

*“When you have expectations about what a medical doctor thinks that you should be doing and how you should be living your life and how much exercise you’re doing and all that sort of stuff, that just adds to the pressure and I think you become unwell.” (female, type 2, late-40s)*

Consequently, “not dwelling” (female, type 2, late 40s) on diabetes, their self-management of diabetes, and diabetes complications was important to promoting mental health and preventing Autistic burnout:

*“It’s a battle every day, so it could be a battle on a number of fronts, including diabetes but in order to not get overwhelmed, I think it’s important for me not to worry about diabetes too much.” (male, type 2, early 70s).*

Participants acknowledged and accepted they could not realise all diabetes self-management activities without contributing to Autistic burnout. This sometimes meant they prioritised activities that minimised Autistic burnout, by promoting their daily functioning and mental health, relative to diabetes self-management:

*“Do I want to get up at six in the morning and do exercise before work? No! I want to sleep, because that sleep is important for me so that I can actually go to work.” (female, type 2, late 40s).*

Being in Autistic burnout impaired diabetes self-management, and its effects on self-management varied between participants. Autistic burnout impacted blood glucose levels because participants were “emotionally distressed” (female, type 2, mid 40s), and could not exercise or prepare healthy meals:

*“…when you burnout the best you can do is take medication…there can’t be any expectation around anything else because in all honesty, you’re just struggling to get through the day, and the struggle is real…” (female, type 2, late 40s).*

For one participant Autistic burnout involved withdrawing from social supports who supported them in self-managing their diabetes. This impacted their ability to complete key self-management tasks such as taking insulin:

*“…when I get that burnout I kind of cut myself off with everyone…and no one knows that I’m not taking care of my diabetes then, so I just keep not taking care of my diabetes…” (female, type 1, early 20s).*

To prevent Autistic burnout by promoting good mental health, and consequently avoid its impacts on diabetes self-management, meeting their Autistic needs was important. This included “limiting interactions” (male, type 2, mid 40s), making downtime, creating “sensory-friendly” (female, type 2, mid 40s) environments where possible, and following routines.

### A Holistic Approach to Diabetes Care is Needed

Most participants informed their health professionals about their autism diagnosis, however, health professionals did not acknowledge participants’ autism while providing diabetes care. Consequently, participants felt their autism and autism-related diabetes needs were dismissed by health professionals:

*“…the majority of the health professionals I see for diabetes do know about my autism diagnosis, but it doesn’t make any difference, the practice still remains the same as it would.” (male, type 2, early 70s).*

When health professionals acknowledged and were sensitive to autism-related needs, this produced positive health care experiences. Participants were more comfortable with and consequently sought out health professionals who understood autism:

*“Seeing my GP is always a good experience because she just gets autism and disability…there have been lots of bad times though.” (female, type 1, early 40s).*

Health professionals, potentially not realising the need to adapt their communication style, provided insensitive and inappropriate advice around diabetes self-management. One participant did not complete aspects of self-management, such as administering insulin, due to the exhaustion they experienced from sensory overload. Their health practitioner suggested this was a personal failing:

*“....I feel like he doesn’t yeah think about how autism’s impact on executive functioning could be part of it, he just thinks time management, it’s my disorganisation...” (female, type 1, early 20s).*

Participants reported health professionals delivered diabetes advice which did not consider or adapt to their learning and communication styles. This impaired their understanding of the advice, “he just tells me a lot of things and sometimes it just doesn’t mean much to me” (female, type 1, early 20s), and receptivity or uptake of diabetes advice:

*“...they present the whole [diabetes self-management] situation in unmanageable portions which just causes me to reject it…” (male, type 2, mid 40s).*

It was particularly valuable to participants when health professionals identified a need to adjust their communication style. A participant who preferred visual learning initially struggled with processing verbally delivered diabetes education; however, when their diabetes nurse educator became aware of their difficulties and modified diabetes education in response, they experienced discernible improvements in their uptake and retention of the knowledge:

*“...somehow I think my diabetes educator understood that I wasn’t understanding things when he just told them to me, so that’s when he started giving me those books and more visual things. He was really, he was really smart.” (female, type 1, early 20s).*

The impact of receiving health care that did not meet their needs sometimes culminated in participants avoiding health care for their diabetes self-management. As the support person of one participant described, “[Autistic people] have to build up courage to go to the doctor” (female, mid 30s). Participants perceived the loss of appropriate diabetes care, and health professionals’ inadequate support more acutely, and felt discouraged from seeking care. Often, to receive health care that met their needs, participants simply wanted health professionals to acknowledge their autism:

*“...if you see someone struggling with their diabetes, instead of telling them off, just talking about it, just saying how is your autism going?” (male, type 1, mid 30s).*

## Discussion

This is the first qualitative study to explore the lived experiences of Australian Autistic adults living with diabetes. Within this study, each participant approached their diabetes uniquely with complex nuances to the way their Autistic experience influenced their diabetes self-management. We found Autistic individuals reported autism to influence various aspects of diabetes self-management including daily tasks like taking insulin and engaging with diabetes healthcare professionals. Our findings on the impacts of Autistic burnout on health management are an important contribution to the field; participants often considered autism as a higher priority than diabetes due to the possibility of Autistic burnout and its impact on diabetes self-management. Consequently, participants tried to avoid dwelling on diabetes and its complications to minimise the existing pressures on mental health they experienced. Health professionals separated participants’ diabetes and autism; given the impacts of Autistic experiences on diabetes self-management, health professionals subsequently provided diabetes care which was mismatched to Autistic needs and experiences and therefore ineffective or difficult to adopt. More broadly, our findings on autism’s influences on diabetes self-management have implications for Autistic adults’ health management in general.

Good patient-provider communication is essential for self-management and shared decision-making where health professionals bring their clinical knowledge, and individuals bring expertise of their lived experience to mutually create diabetes-related health advice (Joosten, et al., 2008; Kashaf, et al., 2017; Nam, et al., 2011). Similar to non-Autistic adults, Autistic adults experienced poor patient-provider communication; unlike the general population, Autistic adults felt uncomfortable sharing their needs due to unsupportive healthcare environments resulting in masking. Participants masked through passively agreeing with the health professional’s advice without raising concerns or challenges they attributed to autism. At times, like other research has reported (Bradshaw, et al., 2019; Nicolaidis, et al., 2015), Autistic adults did not share their autism diagnosis for fear of discrimination and dismissal of needs. This led to poor patient-provider communication and diabetes care that did not meet participants’ needs as their Autistic experience often impacted diabetes self-management.

The importance of the connection between Autistic burnout and diabetes self-management is a key finding of this study. Alongside impacting day-to-day social and occupational life spheres (Higgins, et al., 2021; Raymaker, et al., 2020), this study adds that Autistic burnout affects diabetes self-management and was perceived as more impactful than diabetes burnout. Participants could only perform what they determined as the most basic of diabetes self-management tasks to survive while in Autistic burnout, implying Autistic burnout likely has negative impacts on physical health and its management. The high risk of serious diabetes complications from sustained blood glucose levels outside of target ranges (Smith-Palmer, et al., 2014), underscores the importance of further research investigating the relationship between Autistic burnout and diabetes. It would be important to characterise how key variables, such as duration of Autistic burnout, could impact the self-management of diabetes and other physical health conditions. Autistic burnout may thus be a vulnerable period during which Autistic adults require greater support in managing their diabetes and health.

In this study, we further reinforced knowledge of the barriers Autistic adults experience when accessing healthcare. The sensory environment of clinical spaces, the skills required to navigate complex health systems and the lack of understanding and skills of health professionals working with Autistic adults are well-documented (Bradshaw, et al., 2019; Nicolaidis, et al., 2015). Our study further highlighted the limited skills health professionals have in adjusting their delivery of healthcare for Autistic adults. While upskilling health professionals in diagnosing and recognising autism has rightly been a prominent clinical recommendation from research involving Autistic people (Austriaco, et al., 2019; Bordini, et al., 2015; Garg, et al., 2014; Ghaderi & Watson, 2019), our findings suggests that autism education for health professionals should also emphasise how the Autistic experience impacts Autistic people’s health management, including autism-specific strengths and challenges.

Creating inclusive healthcare environments, which reduce the need to mask, is imperative to improving communication between Autistic adults and their professionals. This will help facilitate the provision of health advice which better meets Autistic adults’ needs and subsequently facilitate shared decision-making (Joosten, et al., 2008; Kashaf, et al., 2017; Nam, et al., 2011). Autism knowledge could further train health professionals in providing post-autism diagnosis support to equip Autistic adults with the knowledge on how autism-specific strengths and challenges influence their diabetes self-management, to facilitate addressing these challenges. This is as the benefits conferred post-diagnosis did not come about organically for some participants and are possibly dependent on formal or informal education.

### Broader Implications and Future Research Directions

Given the barriers Autistic adults faced to adapting to traditional diabetes self-management approaches, our study demonstrates the need for further research in adapting diabetes management, and health management more broadly, to suit Autistic needs and experiences. Prominent health management models which place physical health conditions at the forefront poorly address the approach to diabetes and health management favoured by participants in our study. Future research should thus co-produce models of self-management that are more reflective of Autistic individuals’ priorities. Additionally, health professionals are ideally placed to play a key role in supporting Autistic adults through Autistic burnout, particularly as supportive environments appear to facilitate improved energy levels while experiencing burnout (Higgins, et al., 2021). Therefore, future research should identify ways to equip health professionals in understanding and recognising the signs of Autistic burnout to support Autistic individuals during this period to ensure their physical health management is not entirely neglected in this time. Future research should identify how key variables, such as duration of Autistic burnout, could further impact the self-management of diabetes and other physical health conditions. This could also include creating sick day plans-which for non-Autistic individuals generally revolve around modifications to diabetes self-management when experiencing physical ill health-to implement when an individual is in Autistic burnout.

### Strengths and Limitations

Our research was strengthened by the involvement of an Autistic researcher across all stages of the research. However, we recognise the input of the Autistic researcher in this project lives with type 2 diabetes and our research might have subsequently overlooked nuances in the experiences of Autistic people with type 1 diabetes. Future research should ideally include Autistic researchers, or research advisors, with type 1 diabetes and type 2 diabetes. Recruiting Australia-wide and including non-physical interview methods facilitated recruitment. However, our strategy failed to recruit individuals with an intellectual disability, those with higher support needs or those who were significantly impacted by the financial aspects of diabetes. The limited range of age at autism diagnosis potentially limited our insight into differences in the lived experience of Autistic individuals diagnosed during childhood compared to adulthood. We did not seek confirmation of autism and diabetes diagnoses, relying on self-reports of these conditions, possibly limiting our results. Future research should aim to capture a more diverse population of Autistic adults living with diabetes. Due to the small sample, we could not achieve data saturation as each participant bought new experiences and data. Our understanding of this group’s lived experience increased with each interview and our interview guide changed to reflect new insights that we sought to explore in future research.

## Conclusion

Our research demonstrated that providing diabetes care whilst also meeting Autistic needs is essential; Autistic individuals have unique needs that impact diabetes self-management. It is imperative health professionals understand the impact of Autistic burnout and masking on diabetes self-management. Therefore, creating environments that empower Autistic individuals in chronic disease management is vital to improving the health inequities experienced by this group. Lastly, there is a pressing need for researchers to listen and seek the views of Autistic individuals to tailor supports across all spheres of healthcare, from health advice to health access. Our research has laid the groundwork, but more is sorely needed.

## Footnotes

1 This paper uses identity-first language (i.e., Autistic adult) as preferred by many Autistic adults and their allies (Kenny, et al., 2016).
