# Correction to: “My Autism is Linked with Everything”: at the Crossroads of Autism and Diabetes

**DOI:** 10.1007/s10803-024-06431-1

**Published:** 2024-06-14

**Authors:** Ritwika Vinayagam, Christopher Tanner, David Harley, Shamshad Karatela, Katie Brooker

**Affiliations:** 1 Queensland Centre for Intellectual and Developmental Disability (QCIDD), Mater Research Institute-UQ, The University of Queensland, Level 2, 39 Annerley Rd, South Brisbane, QLD 4101 Australia; 2 The Cooperative Research Centre for Living with Autism (Autism CRC), Level 3, Foxtail Building UQ Long Pocket Campus, 80 Meiers Rd, Indooroopilly, QLD 4068 Australia; 3 UQ Centre for Clinical Research, Royal Brisbane & Women’s Hospital Campus, Building 71/918, Herston, QLD 4029 Australia; 4 School of Pharmacy, The University of Queensland, Level 4/20 Cornwall St, Woolloongabba, QLD 4102 Australia; 5 Australian Institute of Tropical Health and Medicine (AITHM), James Cook University, Building 48 1 James Cook Drive, Douglas, QLD 4811 Australia

**Correction to: Journal of Autism and Developmental Disorders (2023)**

10.1007/s10803-023-06033-3

In the original article, one of the co-author name has been mistakenly published as Shamshad Karatella.

The correct name should read as Shamshad Karatela

This has been corrected in this article.

